# Transcriptomic and Metabolomic Profiling Reveals the Effect of LED Light Quality on Fruit Ripening and Anthocyanin Accumulation in Cabernet Sauvignon Grape

**DOI:** 10.3389/fnut.2021.790697

**Published:** 2021-12-14

**Authors:** Peian Zhang, Suwen Lu, Zhongjie Liu, Ting Zheng, Tianyu Dong, Huanchun Jin, Haifeng Jia, Jingggui Fang

**Affiliations:** Key Laboratory of Genetics and Fruit Development, Horticultural College, Nanjing Agricultural University, Nanjing, China

**Keywords:** light quality, anthocyanin biosynthesis, sugar acid metabolism, volatile substances, WGCNA

## Abstract

Different light qualities have various impacts on the formation of fruit quality. The present study explored the influence of different visible light spectra (red, green, blue, and white) on the formation of quality traits and their metabolic pathways in grape berries. We found that blue light and red light had different effects on the berries. Compared with white light, blue light significantly increased the anthocyanins (malvidin-3-*O*-glucoside and peonidin-3-*O*-glucoside), volatile substances (alcohols and phenols), and soluble sugars (glucose and fructose), reduced the organic acids (citric acid and malic acid), whereas red light achieved the opposite effect. Transcriptomics and metabolomics analyses revealed that 2707, 2547, 2145, and 2583 differentially expressed genes (DEGs) and (221, 19), (254, 22), (189, 17), and (234, 80) significantly changed metabolites (SCMs) were filtered in the dark vs. blue light, green light, red light, and white light, respectively. According to Gene Ontology (GO) and Kyoto Encyclopedia of Genes and Genomes (KEGG) analyses, most of the DEGs identified were involved in photosynthesis and biosynthesis of flavonoids and flavonols. Using weighted gene co-expression network analysis (WGCNA) of 23410 highly expressed genes, two modules significantly related to anthocyanins and soluble sugars were screened out. The anthocyanins accumulation is significantly associated with increased expression of transcription factors (*VvHY5, VvMYB90, VvMYB86*) and anthocyanin structural genes (*VvC4H, Vv4CL, VvCHS3, VvCHI1, VvCHI2, VvDFR*), while significantly negatively correlated with *VvPIF4. VvISA1, VvISA2, VvAMY1, VvCWINV, Vv*β*GLU12*, and *VvFK12* were all related to starch and sucrose metabolism. These findings help elucidate the characteristics of different light qualities on the formation of plant traits and can inform the use of supplemental light in the field and after harvest to improve the overall quality of fruit.

## Introduction

Grape (*Vitis* spp.) is a globally important fruit that is cultivated on all continents. It is one of the most economically favorable fruit crops due to its high flavonoid content, sweet and sour flavor characteristics, and nutritional value and is also an important raw material for winemaking (1). Anthocyanin, sugar, organic acids, and aroma are important agronomic traits that determine berry flavor characteristics and wine quality to a large extent. Flavonoids represent a widespread and common group of natural polyphenols produced by the phenylpropanoid pathway (2). They confer ultraviolet (UV) protection, assist in seed dispersal by attracting dispersers, and act as tissue protectors under pathogen attack or oxidative damage (3). Flavonoids are the main groups of soluble phenolics in grapes as well as the major contributors to the biological activities in products derived from grapes. The organoleptic quality of table grapes, as well as the wine quality, flavor, and stability, greatly depends on the content and composition of the sugars and organic acids in grape berries (4). The various volatile substances in the fruit can further enhance the sensory experience of table grapes and wine (5).

Light is one of the most important factors affecting plant growth and fruit quality formation. Different light intensities, qualities, and photoperiods will significantly affect plant growth and physiological metabolism (6). Experiments involving bagging and different light quality treatment experiments on different fruits, including grapevine, strawberry, apple, pear, tomato, resulted in changes in flavonoids, soluble sugars, the sugar acid ratio, and aromatic components (6–9). It appears that a shorter wavelength (in the range of blue light (420–490 nm), UV-A (315–400 nm), UV-B (280–315 nm), and UV-C (200 nm-280 nm)) usually plays the most prominent role in the accumulation of flavonoids in the fruits by increasing the expression of flavonoid pathway genes (10, 11). Longer wavelengths [in the range of red light (600–700 nm) and far-red light (700–800 nm)] seem to increase the content of soluble sugars in fruits (12). In addition, green light, yellow light may also play a role in altering fruit quality (13). These findings have fostered interest in the use of light-emitting diode (LED) lights in orchards to improve the nutritional value and bioactive compounds of ripe fruit. Most previous studies have focused on the effects of a single light quality on physiological changes in plants, while few reports have compared the effects of different light qualities under the same environmental conditions (7, 14). Furthermore, little is known about the molecular basis of the effect of red, blue, green, and white light on the formation of various quality traits in fruits.

Most higher plants can sense specific light wavelengths spanning from ultraviolet-B (UV-B) to far-red wavelengths. Arrays of photoreceptors, including UV RESISTANCE LOCUS 8 (UVR8) induced by UV-B (15), cryptochromes (CRY1, CRY2, CRY3) and phototropins (PHOT1, PHOT2) that absorb ultraviolet-A (UV-A)/blue light (16, 17), and phytochromes (PHY) that respond to red/far-red light, have been reviewed (18). Under different light environmental conditions, each photoreceptor regulates the development of light morphology by inhibiting the activity of the ring finger ubiquitin E3 ligase CONSTITUTIVE PHOTOMORPHOGENIC 1 (COP1) and SUPPRESSOR OF PHYA (SPA) complex (19). The bZIP transcription factor ELONGATED HYPOCOTYL5 (HY5) is a targeted transcription factor downstream of COP1 that can promote photomorphogenesis (20). HY5 has also been linked to the activation of the R2R3 MYBs and key structural genes of the flavonoid pathway, as well as the accumulation of flavonoids in response to light in *Arabidopsi*s and apple (21, 22). In addition, some members of the phytochrome-interacting factor (PIF) family have been found to interact with phytochromes and participate in anthocyanin biosynthesis by binding to the promoters of related genes (23, 24). Among them, PIF3 can promote anthocyanin accumulation, while PIF4 and PIF5 may regulate *CHS, F3'H, DFR, LDOX, PAP1*, and *TT8* and play a negative regulatory role (25, 26). However, there are relatively few studies on the molecular mechanisms of different light quality signal responses in grapes.

In recent years, advances in high-throughput methods such as transcriptomics and metabolomics have enabled the parallel screening of metabolites and related genes underlying organ coloration in plants. The present study comprehensively evaluated the impact of LED light qualities (red, green, blue, and white) on pre-veraison. “Cabernet Sauvignon” grape clusters. Based on the fruit physiological profiles, transcriptome, metabolome, and WGCNA, the effects of different light qualities on the grape light response, anthocyanins, sugars, and acid metabolism synthesis were explored, and the associated regulatory networks were analyzed. This study provides a theoretical basis for the use of LEDs of different light qualities to supplement light in orchards to improve the quality of grapes.

## Materials and Methods

### Fruit Material and Different Light Quality Treatments

The pre-veraison grape (*Vitis vinifera* L. cv. “Cabernet Sauvignon”) clusters were collected from 7-year-old grapevines that had been planted in the Taiyangcheng Winery (35°74′N, 119°45′E), Rizhao, Shandong, China. Within orchards, 60 clusters with uniform orientation, setting position, tightness, and devoid of damage and external defects were selected for collection. The fruits were immediately delivered to the laboratory and washed 4–5 times with 5% sodium hypochlorite and distilled water. After the moisture on the surface of the fruit had evaporated in the dark, the clusters were assigned to five different groups. The clusters were placed in a constant temperature and humidity incubator (24°C light for 14 h; 20°C dark for 10 h) (MGC-380A-LED, Pulangke, Ningbo, China) with 90–95% relative humidity (RH).

These clusters were placed under white (350–750 nm), red (660 nm), green (530 nm), and blue (450 nm) LED lights (Pulangke, Ningbo, China), the light quantum flux was adjusted using a spectroradiometer (Konica Minolta, Tokyo, Japan), each light was maintained at approximately 50 ± 5 μmol·m^−2^·s^−1^. The control had no light treatment. Each treatment treated 12 clusters as biological replicates. After 8 days of treatment, randomly collected all samples from four light treatment groups and one control group from the clusters. The skin, flesh, and seeds of the fruit were separated and immediately frozen in liquid nitrogen and stored at −80°C.

### Measurement of Organic Acids, Soluble Sugars, and Aromatic Components in the Grape Flesh

After the grape flesh from the different treatments had been ground with liquid nitrogen, extracted three biological replicates for each treatment, the soluble sugar and organic acid contents were determined using ultra-performance liquid chromatography (UPLC, Waters, Milford, MA, USA). The extraction and determination methods were based on the Yang et al. (27). The conditions for separating the soluble sugars were as follows: detector, differential refraction detector; column, Agilent ZORBAXSB-C18 (4.6 × 250 mm, 5 μm); phase, acetonitrile/water = 80/20 (v/v); flow rate, 1.0 ml/min; injection amount, 10 μl; column temperature, 40°C; and analysis time, 20 min. The conditions for separating the organic acids were as follows: detector, diode array detector; column, Thermo Hypersil COLD aQ (4.6 × 250 mm, 5 μm); phase, 0.01 mol/L KH_2_PO_4_ (pH = 2.55)/methanol = 97/3 (v/v); flow rate, 0.5 ml/min; injection amount, 20 μl; column temperature, 25°C; and analysis time, 20 min. The aromatic components were determined using gas chromatography–mass spectrometry (GC-MS, Thermo Fisher, Waltham, MA, USA), and the extraction and determination methods were based on Zheng et al. (28). 3 g material after grinding is dissolved in 3 ml NaCl for determination. One microliter of each sample was injected in split mode (ratio 1:5) with about 17% of injected samples being transported by a carrier gas into a non-polar column (TG-5MS, 30 m, 0.25 mm ID, 0.25 μm film thickness, Thermo Scientific). Compounds were tentatively identified by mass spectrometry analyses: i.e., matching mass spectrum of samples with database in NIST mass spectral library. 3-octanol was used as an internal standard substance.

### Measurement of Total Anthocyanins and Their Components in the Grape Skins

Total anthocyanins were extracted using the methanol–HCl method (29). Three biological repetitions for each treatment, the sample (0.5 g) was ground with liquid nitrogen and incubated in 5 ml methanol containing 0.1% (v/v) HCl overnight in the dark at room temperature. The anthocyanin contents in the samples were measured using the pH differential method, as described by Lee et al. (30). The absorbance of the sample extracts at 520 and 700 nm was measured using a UV-2550 spectrophotometer (Shimadzu, Kyoto, Japan). The anthocyanin components were determined using liquid chromatography–mass spectrometry (LC-MS, Waters, Milford, MA, USA), and the peak area was used to measure the content of each component (31).

### Sample Preparation and Metabolite Extraction

The sample preparation, extract analysis, and metabolite identification and quantification were performed at BGI Co., Ltd (Shenzhen, China), following their standard procedures, which were previously fully described by Manohar et al. (32). Metabolite data analysis was conducted using Compound Discoverer (Thermo Fisher, Waltham, MA, USA).

### RNA Extraction, Illumina Sequencing, and Data Analysis

Fifteen libraries representing the five skin samples and the three replicates were constructed for transcriptome sequencing. Total RNA was extracted using the cetyltrimethylammonium bromide (CTAB) method (33). The libraries were sequenced on an Illumina HiSeq platform, and 150 bp paired-end reads were generated. High-quality clean reads were obtained for further analysis by removing the reads containing adapters, high poly-N, and low-quality reads. Firstly, custom Perl scripts were used to extract the sequence data, in which the base-pair qualities were Q ≥ 20. The filtered reads were mapped to the reference genome (https://www.ncbi.nlm.nih.gov/genome/401) using HISAT2 software (34). Then, raw count data were acquired by Counts and R scripts. Gene expression was estimated by transcripts per million (TPM) using the software Trinity (35). The DESeq2 was used to identify DEGs and the filter condition was |log_2_FC (fold change)| ≥ 1 and the false discovery rate (FDR) <0.05. The DEGs were subjected to GO analysis using the GOSeq R package (corrected *P*-values < 0.05). According to the standard methodology of KEGG, the identified functional genes were mapped, which is useful for further analyses of the networks of the genome.

### Weighted Gene Co-expressed Network Analysis

The correlation of the grape quality traits, including total anthocyanins, soluble sugars, tartaric acid, malic acid, and citric acid, as well as aromatic components, with the transcriptome data was assessed. The highly co-expressed gene modules were inferred from these filtered genes using the R package WGCNA (36). WGCNA network construction and module detection were conducted using an unsigned type of topological overlap matrix (TOM), with a power β of 10 and a branch merge height of 0.85. The module eigengene (the first principal component of a given module) value was calculated and used to evaluate the association of modules with fruit physiological indexes in all samples. Regulatory network diagrams were illustrated using Cytoscape 3.7.1 (37).

### CDNA Synthesis and qRT-PCR

The cDNA was synthesized using a HifairII First Strand cDNA Synthesis SuperMix for qPCR (Yeasen, Shanghai, China). The quantitative real-time PCR (qRT-PCR) comprised 5 μl of Hieff Unicon^®^ Universal TaqMan multiplex qPCR master mix (Yeasen, Shanghai, China), 0.3 μl of each primer (10 μM), 2 μl of cDNA, and 2.4 μl of RNase-free water in a total volume of 10 μl. The reaction was performed using a QuantStudio 6 Flex Real-Time PCR System (Thermo Fisher, Waltham, MA, USA), with the preliminary step at 95°C for 30 s, followed by 40 cycles at 95°C for 5 s and 58°C for 35 s. All biological samples were assayed in technical triplicates. *VvUBQ* (100241514) was used as internal standards and for normalizing the expression. Three biological replicates were performed for all q RT-PCR experiments. The relative gene expression was calculated using the 2^−Δ*ΔCt*^ method. Specific primers used for qRT-PCR are listed in Supplementary Table S1.

### Statistical Analysis

All data (at least three replications) were presented as means with the standard deviation (SD). The mean ± SD values were calculated for each treatment using Microsoft Excel (Microsoft Corporation, Redmond, WA, USA). Statistical analysis of variance (ANOVA) was performed using SPSS 17.0 (SPSS Inc., Chicago, IL, USA) with Duncan's multiple range test at *P* < 0.05.

## Results

### Effects of Different Light Quality Treatments on the Physiological Profile of Grape Berries

**Figure 1:**
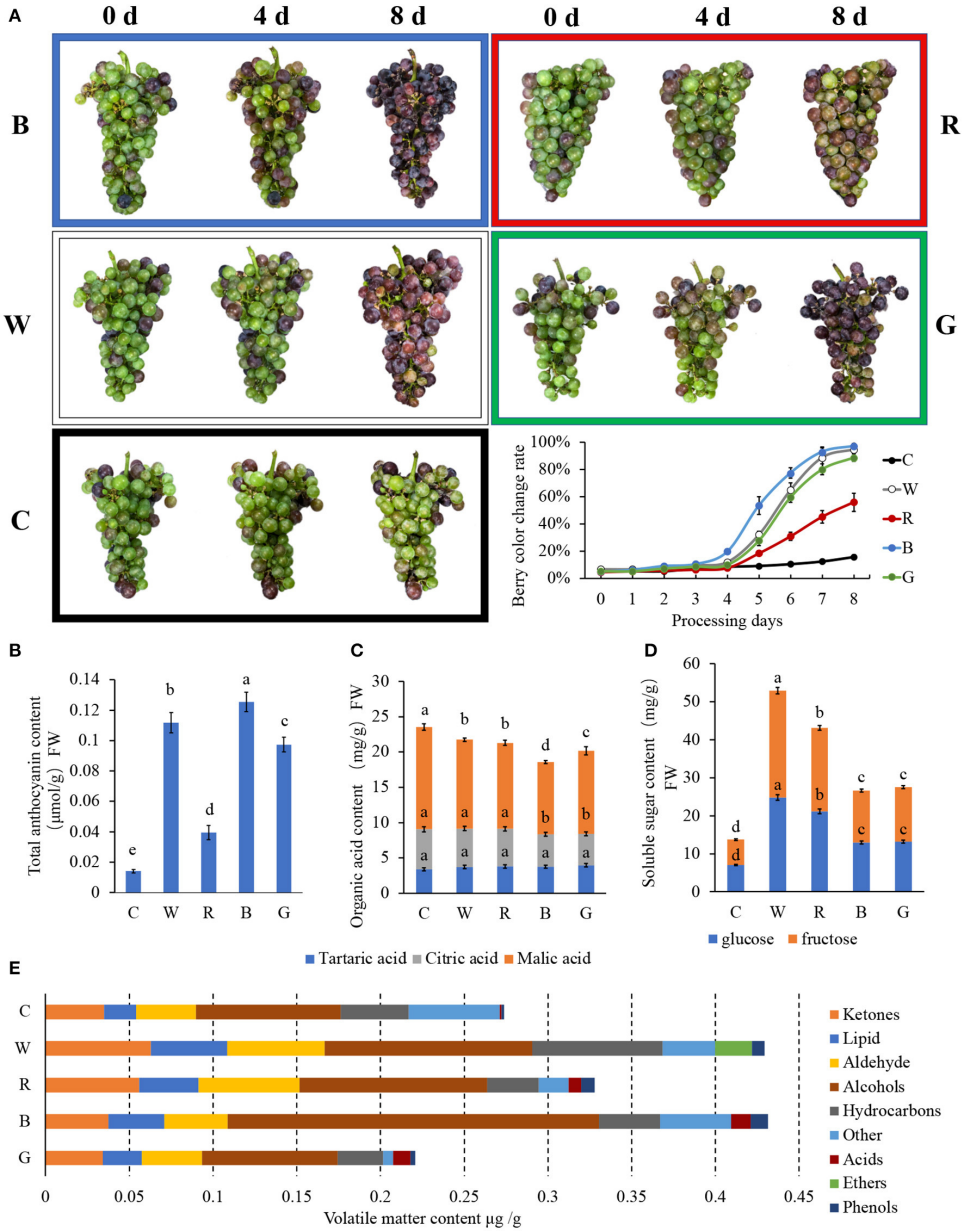
Physiological characteristics of “Cabernet Sauvignon” after different light quality LED treatments. The effects of different light quality treatments on the exterior color **(A)**, soluble sugar **(B)**, organic acid **(C)**, total anthocyanin **(D)**, and volatiles components **(E)** content of “Cabernet Sauvignon.” C: control (no light treatment), B: blue light treatment, R: red light treatment, W: white light treatment, G: green light treatment. Vertical bars represent the standard deviation (SD) of the mean (*n* = 3). Different letters indicate a significant difference at *p* < 0.05, as determined by Duncan's multiple range test.

The changes in the grape berries under different LED light quality treatments are shown in Figure 1A. The rate of berry color change exhibited an S-shaped curve. After 4 d of treatment, 19.7% of the fruits under blue light had begun to color, while there was no significant difference in the others. After 8 d, under blue light, white light, and green light, the grape clusters were almost completely colored, but the degree of coloration of the clusters treated with white light and green light were weaker than that treated with blue light. In addition, only 56.0 and 15.8% of the berries were colored in the red light and control, respectively.

#### Total Anthocyanins Content

Figure 1B shows TAC in the grape skin after 8 d of different light quality treatments. The TAC after blue light treatment was the highest, reaching 0.125 μmol/g. Under white light and green light, the TACs were 0.112 and 0.097 μmol/g, respectively. For the red light treatment and the control, the TACs were lower than that for the blue light treatment, at only 31.5 and 11.2%, respectively.

#### Monomeric Anthocyanins

In order to better understand the anthocyanins and their derivatives in grape skin under different light quality treatments, we determined the content of six major monomeric anthocyanins and their derivatives, including cyanidin-3-*O*-glucoside (Cy), delphinidin-3-*O*-glucoside (Dp), peonidin-3-*O*-glucoside (Pn), petunidin-3-*O*-glucoside (Pt), pelargonidin 3-*O*-glucoside (Pl), and malvidin-3-*O*-glucoside (Mv), in the grape skin subjected to different light quality treatments (Supplementary Table S2).

The content of Cy under blue light was significantly higher than that under white light at 0.883 and 0.135 mg/kg, respectively. Almost all Dp and Cy and their derivatives were detected only after white and blue light treatment, and the content after blue light treatment was significantly higher than that of white light. The contents of Dp after blue and white light treatment were 2.263 and 0.612 mg/kg, respectively, while those of Cy after treatment were 0.135 and 0.883 mg/kg, respectively. The content of delphinidin 3-*O*-(6"-p-coumaroyl-glucoside) in the two groups was the opposite, and its content in the white light group (0.340 mg/kg) was significantly higher than in the blue light group (0.122 mg/kg), and the content detected under green light was similar to that under blue light at 0.127 mg/kg.

Pn and its derivatives were detected in each treatment, but no Pn was detected under white light. The contents of Pn under green light, blue light, red light, and the control were 0.676, 3.565, 0.489, and 0.163 mg/kg, respectively, which were similar to the contents of acetyl anthocyanin [peonidin 3-*O*-(6”-acetyl-glucoside)] at 0.559, 1.487, 0.461, and 0.189 mg/kg, respectively.

Pt and its derivatives were detected in all light treatment groups, but Pt was only detected in the green light group at 0.143 mg/kg. The contents of its derivatives were the highest in the white light group, of which the contents of petunidin 3-*O*-(6”-caffeoyl-glucoside), petunidin 3-*O*-(6”-p-coumaroyl-glucoside), and petunidin 3-*O*-coumaroylglucoside-5-*O*-glucoside were relatively high at 0.743, 0.862, and 0.895 mg/kg, respectively.

Mv is the monomeric anthocyanin that has the largest proportion of total anthocyanins in grapes. Therefore, the change in the Mv content largely affects the change in the TAC in grapes. The Mv detected under white light, green light, blue light, red light, and the control treatments was 5.968, 6.644, 18.234, 0.125, and 0.588 mg/kg, respectively. Of these, five types of acetylated anthocyanins were detected under white light, while only 1–2 derivatives were detected in the other groups. Malvidin 3-*O*-(6”-p-coumaroyl-glucoside) was also detected under white light, green light, blue light, red light, and the control treatments, and its content was 2.583, 5.323, 4.922, 0.250, and 0.425 mg/kg, respectively. In addition, a small amount of Pl (0.143 mg/kg) was only detected under blue light. In the detection and analysis of anthocyanins, it can be found that blue light can promote the formation of various anthocyanins in the grape skin and may play a key role in regulating the formation of Cy and Dp and their derivatives.

#### Organic Acids

Figure 1C shows the three types of organic acids in the flesh. There was no significant difference in the tartaric acid content among the different treatments. The content of citric acid under blue light and green light was slightly lower than under white light, red light, and the control. As for malic acid, the highest content was 14.5 mg/g in the control, followed by the white light, red light, blue light, and green light at 12.6, 12.2, 10.2, and 11.7 mg/g, respectively. Different light qualities can affect the content of malic acid and citric acid in the fruit, which in turn affects the content of total organic acids.

#### Soluble Sugar

As observed in previous studies, the content of glucose and fructose in the grape flesh were similar, while the content of sucrose was minimal (Figure 1D). With fruit ripening, anthocyanins accumulated in the skin, the organic acid content decreased, and the soluble sugar content increased. However, it is interesting that the soluble sugar content of the flesh treated with blue light (26.6 mg/g) and green light (27.5 mg/g), which seemed to be more mature based on the degree of skin coloration, was much lower than that of the grape berry treated with white and red light. The soluble sugar contents under white light and red light were 52.9 and 43.1 mg/g, respectively. The lowest soluble sugar content was detected in the control at only 13.7 mg/g.

#### Volatile Substances

GC-MS was used to determine the volatile substances (Supplementary Table S3), and the quantitative analysis of various volatile substances in all samples was completed in 42.44 min. According to the base peak chromatogram (BPC) and quantitative calculation results (Figure 1E), the total volatile substances under green light were the lowest at 0.221 mg/g. White light had the highest and the most similar volatile substance content to blue light at 0.429 and 0.431 mg/g, respectively, while the red light and the control obtained values of 0.328 and 0.274 mg/g, respectively.

The volatile substances were classified into acids, alcohols, aldehydes, ethers, hydrocarbons, ketones, lipids, phenols, and others for further analysis. Certain differences in the composition categories of the aromatic substances were detected in each treatment group. Among them, the alcohols and phenols in the fruit following blue light treatment were significantly higher than in the other groups, and the hydrocarbons, esters, and acids after white light treatment were also significantly higher than in the other groups. Ketones and aldehydes increased significantly under the red light and white light treatments, whereas the other treatments were comparable to the control.

The isomers between (E)-2-hexen-1-ol and trans-2-hexenol were the most abundant substance in each treatment group. The former was only detected in the dark treatment, whereas the latter process was detected under the different light qualities. Moreover, the content of trans-2-hexenol in each group was higher than the content of (E)-2-hexen-1-ol in the control. The trans-2-hexenal content reached 0.017 mg/g under the white light treatment, followed by the red light and blue light treatments, and relatively less trans-2-hexenal was detected under green light. In addition, caryophyllene (a terpene) was detected only under blue light. We also found a large number of volatile substances with different characteristics, and these may be produced by different light quality treatments, resulting in differences in aromatic and some other properties.

### Metabolome Analysis of Grape Skin After Different Light Quality Treatments

**Figure 2:**
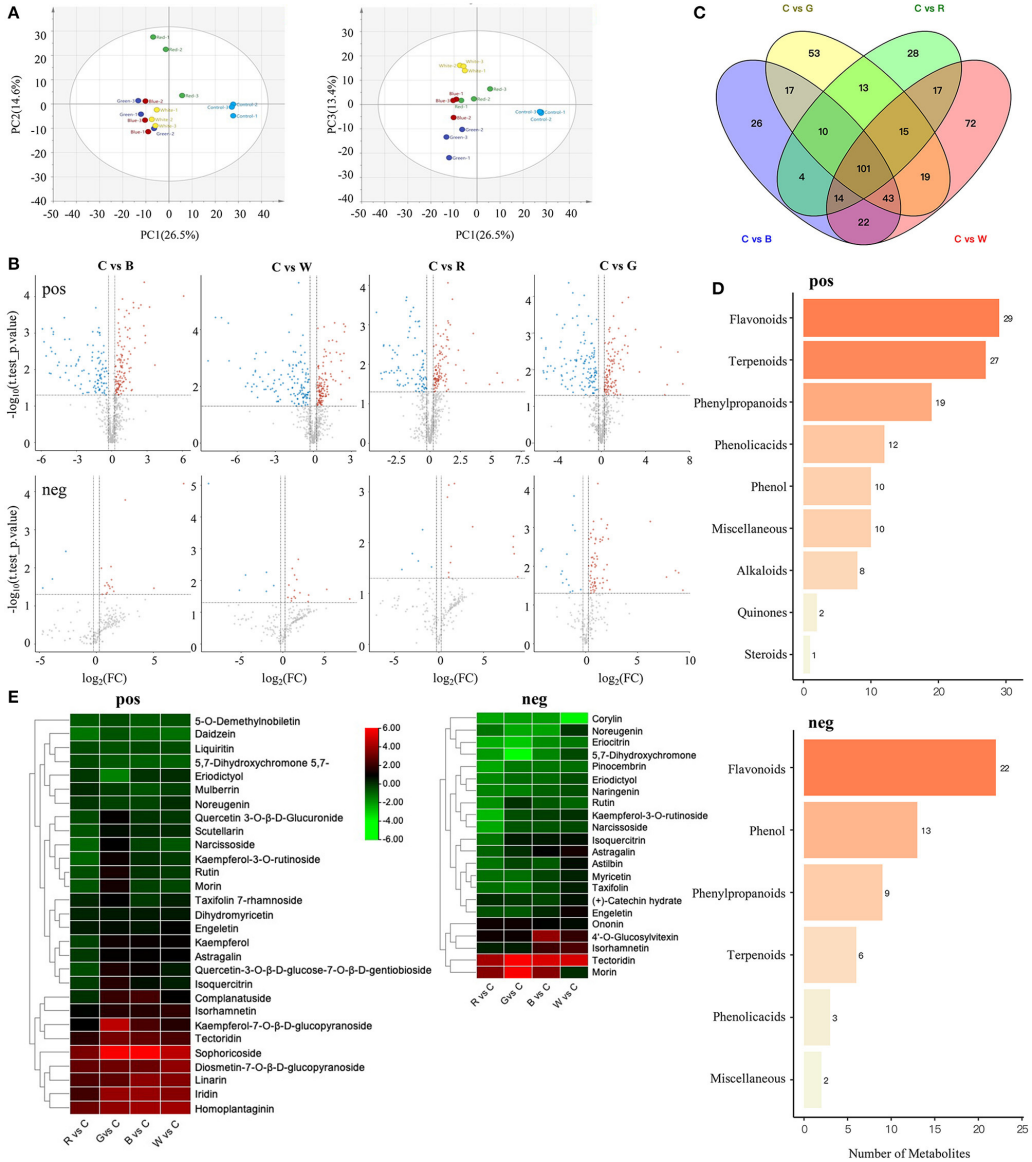
Analyze the metabonomic data of grape berries treated with different light quality. **(A)** PCA score plot metabolite profiles from different treatment groups. **(B)** Volcano plots of significantly changed metabolites (SCMs) between C vs. B, C vs. G, C vs. R, and C vs. W. The first line is pos mode, the second line is neg mode. **(C)** Venn diagram of SCMs. **(D)** KEGG classification of the measured pos and neg metabolites. **(E)** Cluster heatmap of flavonoids and anthocyanins between C vs. R, C vs. G, C vs. B, and C vs. W. C: control (no light treatment), B: blue light treatment, R: red light treatment, W: white light treatment, G: green light treatment, pos: positive mode, neg: negative mode.

In order to clarify the material basis of different light qualities on the color change of the skin, we profiled the metabolome of the five different treatment samples (Supplementary Figure S1). From the principal component analysis shown in Figure 2A, PC1 and PC2 separated the blue light and control samples; PC1 and PC3 separated each group of samples; and the scores of PC1 to PC3 were 26.5, 14.6, and 13.4% respectively, suggesting that the metabolite profiles of these five samples were obviously distinct. The metabolite groupings of the different sample groups were then explored. We detected 838 compounds (including 678 in positive mode, 213 in negative mode, and 53 shared between the two modes) that were grouped into nine classes (Supplementary Table S4). As expected, significantly higher numbers of metabolites (including positive and negative mode) were differentially accumulated between the treatment and control, including (221, 19), (254, 22), (189, 17), and (234, 80) SCMs in C vs. B, C vs. G, C vs. R, and C vs. W, respectively (Figure 2B).

Comparative analysis of the five groups of SCMs among the C vs. B, C vs. G, C vs. R, and C vs. W samples resulted in 101 common metabolites (Figure 2C). The top enriched terms among the SCMs detected for all the compared samples were flavonoids, of which 29 and 22 were detected in the positive and negative mode, respectively (Figure 2D). The combined positive and negative mode results were used to produce clustering heatmaps of 39 flavonoids and anthocyanins (Figure 2E). The contents of some flavonoid precursors, such as 5-*O*-demethylnobiletin, 5,7-dihydroxychromone, and liquiritin, showed significant downregulation in all replicates. In the negative mode, the detected metabolites were mainly flavonoids, and most of them showed significant downregulation, and the contents of flavonoids such as rutin were similarly downregulated in all three replicates. In the positive mode, compared with the control, the content of most anthocyanins under blue light, green light, and white light was significantly upregulated, while in the red light group, only sophoricoside, homoplantaginin, iridin, linarin, and other few anthocyanins were significantly upregulated, the contents of which were lower than in the other three treatments on average.

### DEGs and Transcriptome Analysis of Grapes After Different Light Quality Treatments

**Figure 3:**
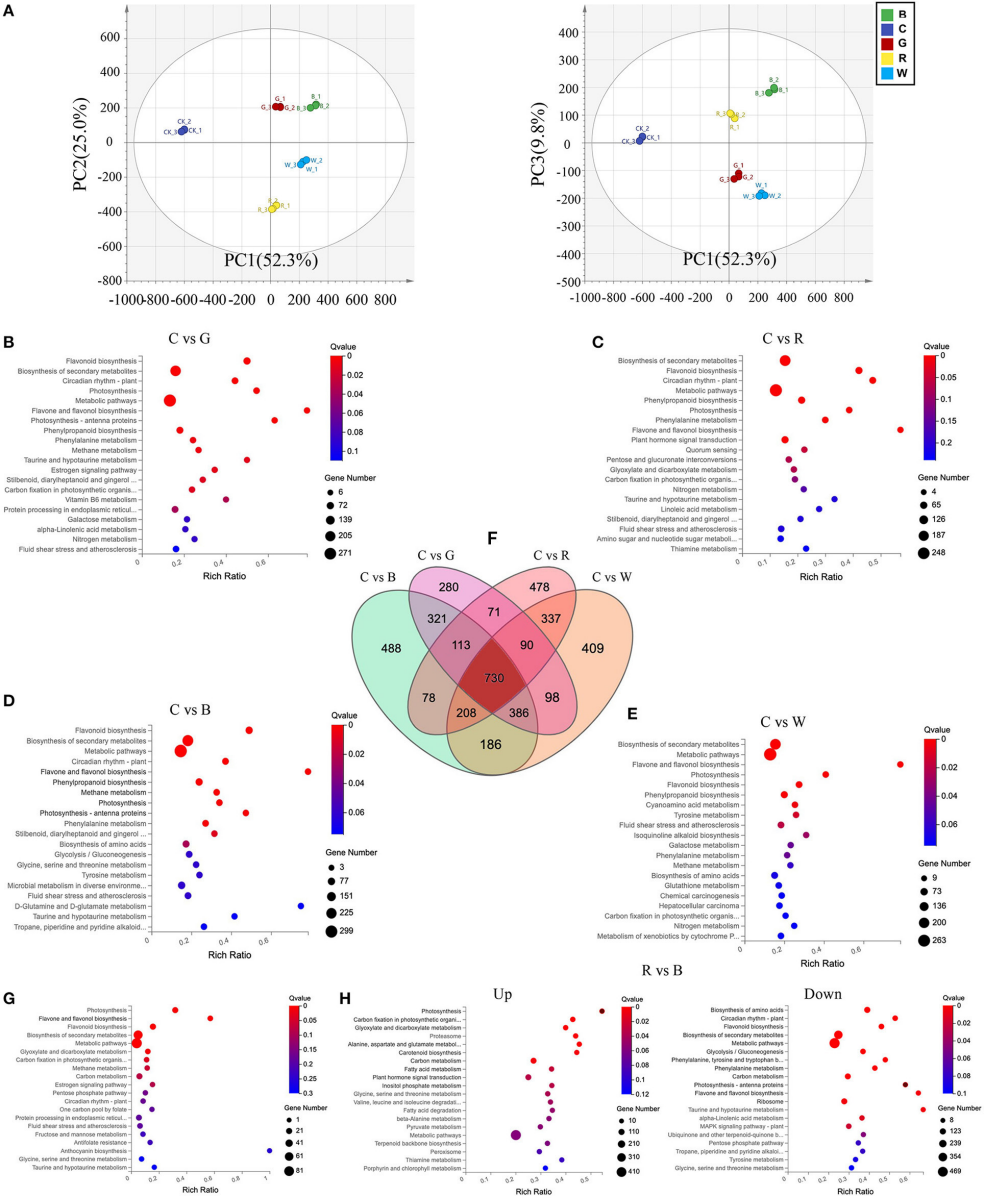
Differential expressed genes (DEGs) in Cabernet Sauvignon after different light quality treatments. **(A)** Principal component analysis of the different light quality treatment based on the gene expression profiles. **(B-E)** KEGG enrichment analysis of the DEGs between C vs. G **(B)**, C vs. R **(C)**, C vs. B **(D)**, and C vs. W **(E)**. **(F)** Venn diagram showing the shared and unique DEGs between the four compared groups of peel samples. **(G)** KEGG enrichment analysis of the C vs. B, G, R, and W 730 DEGs. **(H)** KEGG enrichment analysis of the up-regulation and down-regulation DEGs between R vs. B. C: control (no light treatment), B: blue light treatment, R: red light treatment, W: white light treatment, G: green light treatment. DEGs were identified with the following parameters: *p*-value ≤ 0.05 and |log_2_FC (fold change) | ≥ 1.

Overall, the mapped reads enabled the identification of a total of 23,410 genes with expressed TPM > 0 in at least 1 of 15 examined samples (Supplementary Figure S2). Principal component analysis (PCA) was also conducted on these transcripts (Figure 3A), and the samples under the different light quality treatments could be clearly separated by PC1 and PC2, which together explained 77.3% of the total variation. Only 9.8% of the variation could be explained by PC3. Volcano plots were drawn to reflect the differential gene distribution between each light quality treatment group and the control. There were 2510, 2444, 2105, and 2089 DEGs for C vs. B, C vs. W, C vs. R, and C vs. R, respectively. The top enriched KEGG terms contributed by these DEGs were biosynthesis of secondary metabolites, flavonoid biosynthesis, flavone and flavonol biosynthesis, photosynthesis, metabolic pathways, and others (Figures 3B–E). The differential genes in this experiment were classified into three groups, including molecular function, cellular component, and biological process, by GO classification and were plotted (Supplementary Figure S3).

In the biological process category, there were 2707 (23.0%), 2547 (23.3%), 2145 (23.4%), and 2583 (23.3%) DEGs that were associated with the metabolic process between C vs. B, C vs. G, C vs. R, and C vs. W, respectively. It can be seen that different light quality treatments greatly affected the number of DEGs involved in the metabolic process in grape cells, but the proportion of DEGs involved in metabolism was similar to that of the total DEGs. By clustering the four DEG lists of C vs. B, C vs. W, C vs. R, and C vs. R, we identified the conserved genes that were constantly differentially expressed between the different light quality treatments. We obtained a total of 730 DEGs across the four comparison groups (Figure 3F), suggesting that these core conserved genes may be associated with the response of grape skin to different light qualities. We then enriched the KEGG pathways contributed by these 730 DEGs, which were mainly photosynthesis and biosynthesis of flavonoids and flavonols, with the richness ratio was 31.8 and 57.1%, respectively. All genes enriched in these two pathways were upregulated (Figure 3G). It evidenced that no matter what kind of light qualities can affect photosynthesis and the biosynthesis of flavonoids and flavonols in grape berries, and the number of related DEGs among the blue light, green light, and white light groups was significantly greater than in the red light group.

In order to understand the effect of red light and blue light on the difference of grape, we enriched the DEGs that were up-regulated and down-regulated after red light and blue light treatments (Figure 3H). The up-regulated genes were enriched in photosynthesis, carbon fixation in photosynthetic organism and other related pathways, down-regulated genes enriched pathways such as flavonoid biosynthesis, photosynthesis-antenna proteins, flavonol and flavonol biosynthesis, and others.

### Identification of WGCNA Modules Associated With Fruit Ripening Physiological Traits Under Different Light Quality Treatments

**Figure 4:**
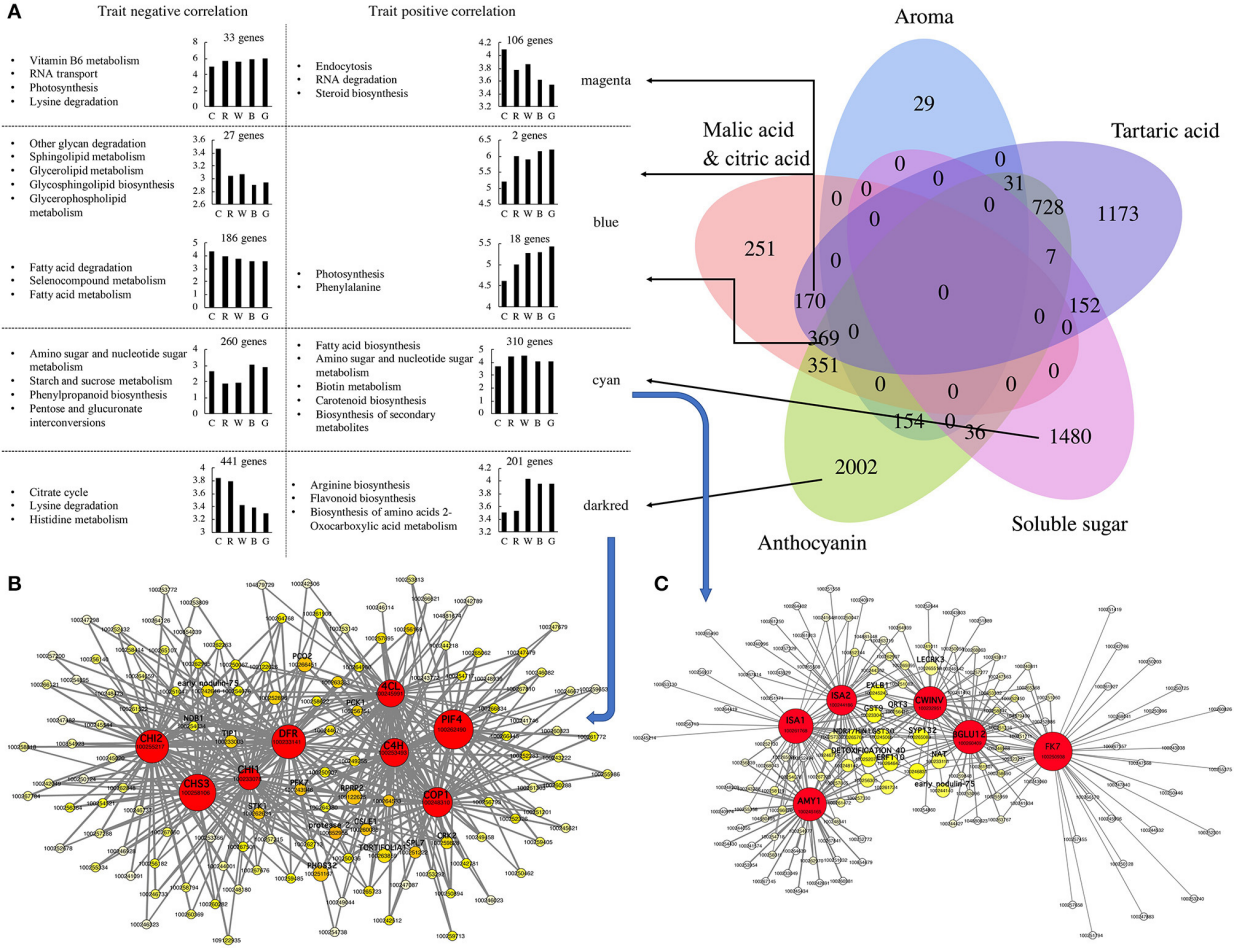
Based on WGCNA (weighted gene co-expression network analysis) to screen anthocyanins, soluble sugars, organic acids and aroma synthesis and metabolism hub genes. **(A)** Construct a Venn diagram based on hub genes screened by anthocyanins, soluble sugar, tartaric acid, aroma, and malic acid and citric acid. Perform KEGG enrichment analysis based on the hub genes in different blocks of Venn diagram, count the number of hub genes that are positively and negatively correlated with the target trait, and draw a histogram of different correlations. Cytoscape representation of co-expressed genes in modules “darkred **(B)** and “cyan” **(C)**. Hub genes are in circle. Each node represents a gene, the color of the circle indicates its connectivity, the size of the node represents the number of connected genes. C: control (no light treatment), B: blue light treatment, R: red light treatment, W: white light treatment, G: green light treatment.

A WGCNA was performed on 23410 genes, leading to the identification of 27 modules (Supplementary Figures S4, S5A). These modules could be clustered into two main branches (one for two modules and another for 25 modules) (Supplementary Figure S5B). The TCA was highly associated with the darkturquoise, purple, blue, and darkred modules, with the first two modules being positively correlated and the latter two modules being negatively correlated. Glucose, fructose, and total sugars were significantly positively and negatively correlated with the green/cyan and darkolivegreen modules, respectively. Organic acids (malic acid, citric acid, and tartaric acid) and total acids were significantly correlated with the blue, magenta, and purple modules, while the correlation between tartaric acid and the other three items was quite different. In addition, aromatic substances were significantly positively correlated with the cyan, darkturquoise, and green modules (Figure 4, Supplementary Figures S5C, S6).

According to the gene significance (GS) and module membership (MM) values of each gene in the different modules, 6933 hub genes related to fruit anthocyanins, soluble sugars, organic acids, aromatic substances, and other traits were screened out. Among them, the number of hub genes related only to anthocyanin content traits reached 2,002, among which the darkred module was the largest, accounting for 642 genes (201 positive correlations and 441 negative correlations). The number of hub genes that were only related to soluble sugar was 1,480, of which the cyan module accounted for 570 genes (310 positive correlations and 260 negative correlations) (Figure 4A). Through further enrichment, we obtained eight and six hub genes in the darkred and cyan modules, respectively, and 541 and 270 edge genes that were highly related to these two groups of hub genes, respectively. Among the eight hub genes related to anthocyanins, *VvC4H* (100253493), *Vv4CL* (100245991), *VvCHS3* (100258106), *VvCHI1* (100233078), *VvCHI2* (100255217), and *VvDFR* (100233141) are all key genes in the anthocyanin biosynthesis pathway and are all upstream genes of the biosynthesis pathway, except for *VvDFR* (Figure 4B). The other two hub genes, *VvPIF4* (100262490) and *VvCOP1* (100248310), are related to the light reaction. Among the pivotal genes related to soluble sugars, *VvISA1* (100261768), *VvISA2* (100244186), *VvAMY1* (100245165), *VvCWINV* (100232951), *Vv*β*GLU12* (100260409), and *VvFK12* (100250938) were all related to starch and sucrose metabolism (Figure 4C). *CWINV* (cell wall invertase) is an extra-cellular enzyme that catalyzes the cleavage of sugar from sucrose into glucose and fructose, and *FK* (fructokinase) efficiently converts fructose into fructose-6-phosphate (Supplementary Figure S7). It may be suggested that the anthocyanin and sugar contents in the fruit were significantly correlated with these hub genes and their edge genes, and their synthesis was greatly regulated at the transcriptional level under different light quality treatments.

To assess whether the expressed transcripts were quantified correctly, five transcripts were selected and analyzed by qRT-PCR using the primers listed in Supplementary Table S4. The results showed that the relative expression and TPM for each gene were significantly correlated (Supplementary Figure S8), confirming the results obtained by RNA-seq analysis.

### Effects of Different Light Qualities on Anthocyanin Biosynthesis and Sugar Acid Metabolism in Grape Berries

#### Light Reaction and Anthocyanin Biosynthesis

**Figure 5:**
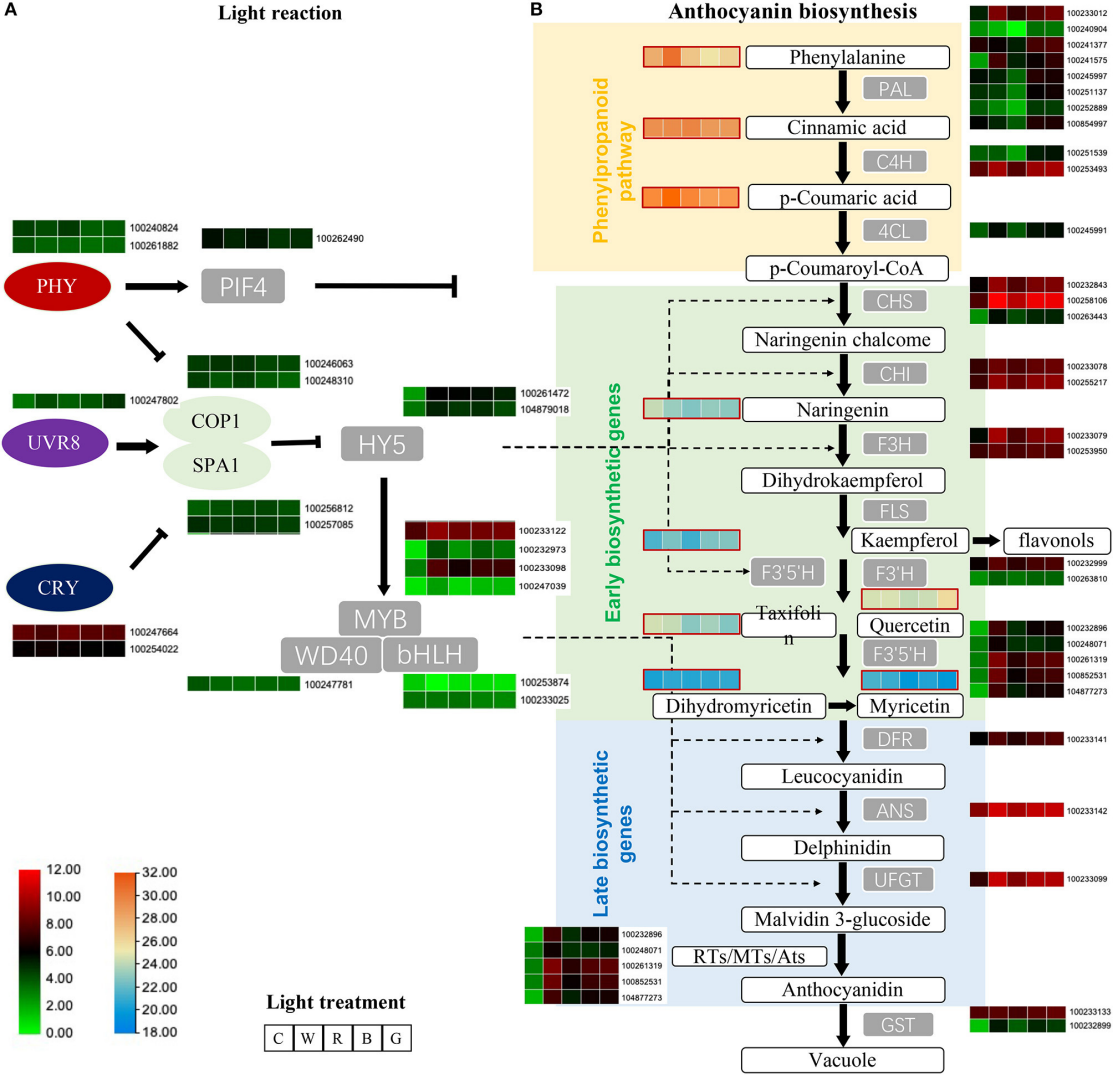
Employ transcriptome and metabolome to explain the differences in light reaction **(A)** and anthocyanin biosynthesis **(B)** pathway genes and metabolites after different light quality treatments. The heatmap displays the expression levels of the genes (average of TPM value) and metabolite [average of log_10_(area value)], from left to right are C, W, R, B, and G. The panel shows in the lower left corner, respectively, indicate the amount of transcriptome (green to red) and metabolome (blue to orange). C: control (no light treatment), B: blue light treatment, R: red light treatment, W: white light treatment, G: green light treatment.

The combined transcriptomics and metabolomics data elucidated the changes in the light reaction and the entire anthocyanin biosynthesis pathway after different light quality treatments. Among the three types of receptor genes upstream of the light reaction pathway, namely *VvPHY, VvUVR8*, and *VvCRY*, the expression trend of *VvPHY* (100240824, 100261882) was negatively correlated with TAC, and the expression of *VvUVR8* (100247802) after different light quality treatments was significantly higher than that of the control, with the green light group exhibiting the greatest expression of *VvUVR8*. The expression level of *VvCRY* (100247664, 100254022) under red light treatment was significantly higher than that under the green light and blue light treatments (Figure 5A).

The expression of the three key transcription factors *VvPIF4* (100262490), *VvHY5* (100261472, 104879018), and *VvMYB* (100233122, 100232973, 100233098, 100247039), which are downstream regulators of the flavonoid pathway, differed significantly from the control. *VvPIF4* was significantly downregulated compared to the control, and the different treatments were negatively correlated with TAC, while two VvHY5 genes were higher than the control after light treatment, the expression level was highest after white light treatment, and green light group was the lowest after light treatment. *VvMYB90* (100233098) and *VvMYB86* (100247039) were also higher than the control after light treatment, the expression level of white light were the highest, followed by blue light and green light, and the expression level after red light treatment were relatively low.

Among the phenylpropanoid pathway upstream of the flavonoid biosynthesis pathway genes, only *VvPAL* (100233012, 100241575) were significantly up-regulated after different light quality treatments compared to the control. Others such as *VvPAL* (100241377, 100245997, 100251137, 100252889, 100854997, 100240904). *VvC4H* (100251539, 100253493, 100267215), and *Vv4CL* (100245991) all showed the lowest gene expression level after red light treatment, and lower than the control, while the expression level under blue light and green light treatment were equivalent and the highest. The content of metabolites of this pathway, phenylalanine, cinnamic acid, and p-coumaric acid, were relatively high in the metabolites related to the anthocyanin synthesis pathway. Among them, phenylalanine and p-coumaric acid changed in the same trend after different treatments, that is, the content of white light treatment was relatively highest, and the content of blue light treatment was the lowest and lower than the control. P-coumaric acid was significantly downregulated in the blue light and green light groups, and the content were significantly increased after red light and white light treatments.

All genes of the flavonoid biosynthesis pathway (*VvCHS, VvCHI, VvF3H, VvF3'H, VvF3'5'H, VvDFR, VvANS, VvUFGT*) were significantly upregulated after light treatment compared with the control, and were consistent with the change trend of TAC.

The metabolome detected six metabolites related to the flavonoid Early biosynthetic pathway (naringenin, kaempferol, taxifolin, quercetin, dihydromyricetin, myricetin). Only kaempferol and dihydromyricetin showed a similar trend to TAC, the six metabolites are relatively less after red light treatment. In addition, the changes in the content of taxifolin and myricetin after different treatments were similar, that is, the content of the light treatment is less than that of the control, and the content of the red light and the green light treatment is the least.

Overall, our results suggest that light can regulate light signal transcription factors, thereby regulating the key functional genes of the flavonoid synthesis pathway and promoting the synthesis of flavonoids (Figure 5B).

#### Sugar and Acid Metabolism

Metabolomics and qRT-PCR were used to analyze the changes in sugar and acid-related genes and metabolites in the grape flesh (Supplementary Figure S7). The metabolite grouping showed that the sucrose content in the grape flesh of each group was relatively low. Among them, the content in the red light group was the highest and the content in the blue light group was the lowest, and a similar trend was observed with the glucose content. G-6-P (glucose-6-phosphate) and F-6-P (fructose-6-phosphate) showed the highest content in the control group, while the former had the lowest content in the green light group.

The expression levels of the key genes *VvSPS, VvSPP*, and *VvSS* for sucrose synthesis and metabolism were significantly upregulated compared to the control, while the downstream *VvIV* genes were all downregulated and their expression levels were opposite to that of the glucose and fructose content in the flesh. FK and HXK (hexokinase) are the key genes for the phosphorylation of fructose and glucose, respectively. The expression of *VvFK1* and *VvHXK2* was downregulated compared with the control, while the expression trend of *VvFK2* and *VvHXK1* was the opposite. The expression of most of the acid metabolism and transport genes, including *VvPK1, VvPK2, VvME2, VvMDH1, VvMDH2, VvCS2, VvALMT1, VvALMT2*, was upregulated compared with the control, and only the expression of *VvCHX* was significantly downregulated compared with the control. The content of tartaric acid, citric acid, and malic acid measured in the metabolome was similar to the results measured by UPLC.

### Modulation of Genes Related to Grape Ripening After Different Light Quality Treatments

To confirm the effect of different light quality treatments on anthocyanin biosynthesis and fruit sugar and acid metabolism in the veraison of “Cabernet Sauvignon,” further examination of the transcriptomics analysis results was needed. qRT-PCR was used to measure the relative expression levels of the key genes in the light reaction, anthocyanin biosynthetic pathway, and sugar and acid metabolism pathway in the grape flesh and skin (Supplementary Figure S9). In the grape skin, the expression levels of *VvCOP2* and *VvSPA2* were significantly downregulated following light treatment compared with the control, and the red light group in the four treatment groups had the highest level of expression. The expression levels of the transcription factors *VvHYH, VvHY5*, and *VvMYB90* were significantly upregulated compared with the control under light treatment and were positively correlated with the content of anthocyanins in the pericarp, while the expression trends of *VvPIF3.1, VvPIF3.2*, and *VvPIF4* were the opposite. The expression levels of *VvC4H* in the blue light and green light groups were significantly higher than that of the control, while the white light and red light treatments demonstrated the opposite. The expressions of other flavonoid biosynthesis genes, including *VvPAL, Vv4CL, VvCHS, VvCHI, VvF3H, VvF3'H, VvF35'H1, VvF35'H2, VvDFR, VvANS, VvUFGT*, and *VvGST1*, were significantly upregulated compared with the control.

In the flesh, the expression levels of the light-responsive transcription factor genes *VvHYH, VvHY5, VvPIF3.1, VvPIF3.2*, and *VvPIF4* differed from those in the skin. *VvHY5* contrasted completely with the anthocyanin content in the skin, and *VvPIF3.1, VvPIF3.2*, and *VvPIF4* all had the highest expression levels under the blue light treatment, followed by the control, while their expression levels under green light, white light, and red light were relatively low. The expression of the aromatic substance synthesis-related genes *VvECar* and *VvQR* was also relatively high under the blue light treatment but low under the other treatments. However, the expression of *VvEGS* in each group was not significantly different (Supplementary Figure S10).

## Discussion

The visible light spectrum ranges between 400 and 710 nm and is subdivided into blue (400–495 nm), green (495–570 nm), yellow (570–590 nm), and red (590–710 nm) light. Previous studies have shown that certain differences exist in the signal receptors and transmission methods for different light qualities in plants (15, 17, 19). It is generally believed that the combination of blue and red light enhances the absorption of photosynthetic pigments, which is conducive to the accumulation of biomass. The former is conducive to the accumulation of chlorophyll and increases the chloroplast a/b ratio, which promotes the accumulation of flavonoids and stem elongation, while the latter has a different effect (38). When fruit-bearing plants are treated with different light qualities, the detached fruit quality characteristics will differ. For example, in blueberry, strawberry, and grape, blue and red light significantly promote the accumulation of anthocyanins and increase the soluble sugar content in the fruits (8, 39, 40). For postharvest non-respiratory fruits (such as blueberries), blue light can increase the content of anthocyanins and reduce the content of soluble solids (TSS), while red light can have the opposite effect (14). The results of this study also found that blue light and red light have different functions on postharvest pre-veraison grapes. The former can promote the accumulation of various anthocyanins such as Mv and Pn in the skin, while the latter can increase the soluble sugar content in the fruit. Transcriptome enrichment results showed that blue light can up-regulate pathway genes such as flavonoid biosynthesis, photosynthesis-antenna proteins, flavonol and flavonol biosynthesis, while red light up-regulates pathway genes such as photosynthesis, carbon fixation in photosynthetic organism. For this reason, we suggested that red light can promote the photosynthesis of the skin thereby increasing the soluble sugar content in the fruit.

In addition to anthocyanins and soluble sugars, the results of this study also show that different light qualities variously influence fruit acid, aroma, and other quality traits. In strawberry, red light and blue light can promote the synthesis of terpenes and aromatic alcohol substances in the fruit, while blue light can reduce the acid content in the fruit (41, 42). The results of this study confirmed this, with blue light and red light having different effects on the flavor composition of grape berries after harvest.

**Figure 6:**
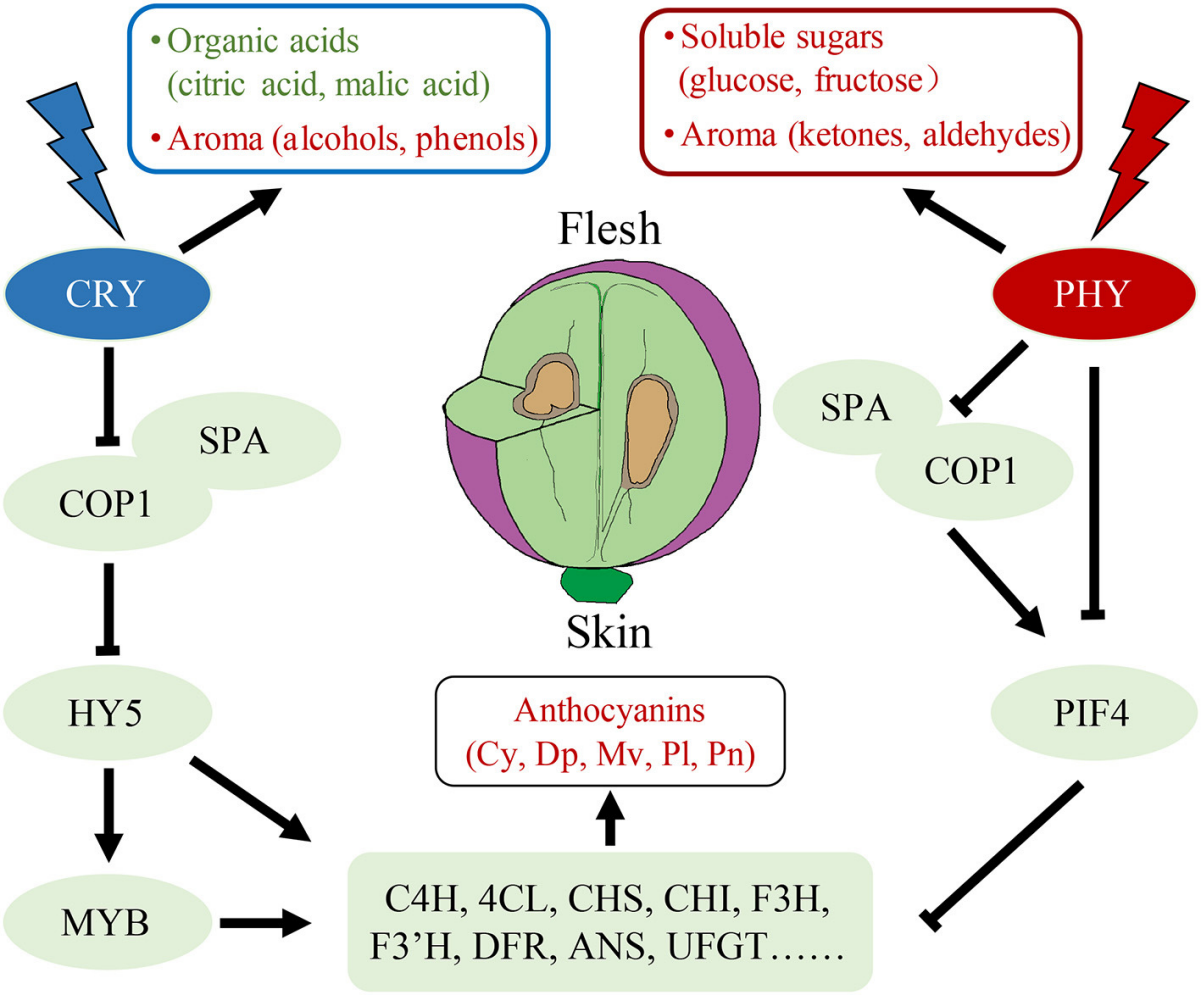
A model of fruit quality traits formation under red light and blue light treatments. Red represents up-regulation while green represents the down-regulation. Cy: cyanidin-3-*O*-glucoside, Dp: delphinidin-3-*O*-glucoside, Mv: malvidin-3-*O*-glucoside, Pl: pelargonidin 3-*O*-glucoside, Pn: peonidin-3-*O*-glucoside.

WGCNA has been used in numerous species to elucidate the interaction between physiological traits and gene expression (37, 43). WGCNA was used herein to evaluate transcript expression and trait changes under different light quality treatments, and two modules, namely darkred and cyan, which were highly correlated with anthocyanins and sugars, respectively, were screened out. In the darkred module, we screened six key functional genes in the anthocyanin synthesis pathway and two light signal response genes *VvCOP1* and *VvPIF4*. Among them, the expression of the transcription factor *VvPIF4* had a significant negative correlation with anthocyanin content and was positively correlated with the red light receptor gene *VvPHY*. In a report on *Arabidopsis*, PIF5 negatively regulated red light-induced anthocyanin biosynthesis and participated in many pathways regulated by PIF4 (25); however, the PIF5 homologous gene has not been identified in grapes (24). Therefore, it can be inferred that when the fruit receives red light, *VvPHY* may inhibit the anthocyanins biosynthesis genes (including *VvC4H, Vv4CL, VvCHS, VvCHI, VvF3H, VvF3'H, VvDFR, VvANS, VvUFGT*, etc.) by directly regulating the expression of *VvPIF4* or promoting the expression of *VvPIF4* through *VvCOP1* and *VvSPA* complex (Figure 6). HY5/HYH, another key transcription factor in the light response pathway, is positively correlated with anthocyanin content (20, 21, 26). We found that the expression level of *VvHY5* was positively correlated with the content of anthocyanins, but the correlation between *VvHYH* and anthocyanin content was poor. It can be speculated that blue light may positively regulate the accumulation of anthocyanins in the pericarp through *VvHY5*.

Different types of anthocyanins are promoted by blue light in different species. For example, delphinidin-3-*O*-glucoside and malvidin-3-*O*-glucoside increased the most in bilberry and grapes after blue light treatment, respectively (44, 45), whereas cyanidin-3-*O*-glucoside increased the most in apple and radish (9). The results of this study indicate that red light may inhibit the synthesis of the most abundant anthocyanin malvidin-3-*O*-glucoside and its derivatives in grapes, while blue light not only greatly promotes the accumulation of malvidin-3-*O*-glucoside but also promotes peonidin-3-*O*-glucoside. Therefore, we speculate that the types of anthocyanins promoted by blue light are not specific.

## Conclusion

In the present study, we used metabolomics and transcriptomics to study the effects of red light, green light, blue light, white light, and no light on the quality traits (anthocyanin components, soluble sugars, organic acids, aromatic substances, and their associated metabolic pathways) of grape berries. Using WGCNA, we screened out modules and their hub genes that were highly related to anthocyanins and soluble sugars, ultimately constructing a model of fruit quality trait formation under red and blue light. These results are helpful for understanding the effects of different light qualities in the formation of plant traits and can inform light supplementation in the field and after harvest to improve the overall quality of the fruit.

## Data Availability Statement

The datasets presented in this study can be found in online repositories. The names of the repository/repositories and accession number(s) can be queried in the NCBI database under the accession numbers: PRJNA769235 (SRR16382595, SRR16382594, SRR16382593, SRR16382592, SRR16382591, SRR16382590, SRR16382589, SRR16382588, SRR16382587, SRR16382588, SRR16382587, SRR16382588, SRR16382587, SRR16382583, SRR16382594, SRR16382593, SRR16382592, SRR16382581).

## Author Contributions

PZ, ZL, and JF conceived and designed the experiments. PZ, ZL, TZ, TD, HJin, HJia, SL, and JF performed the experiments. PZ, ZL, and TZ analyzed the data. PZ wrote the manuscript. All authors read and approved the manuscript.

## Funding

This work was supported by National Key Research and Development Program (2019YFD1000101 and 2019YFD1001904), Jiangsu Agriculture Science and Technology Innovation Fund (CX(18)2008), and Jiangsu Agricultural Industry Technology System (JATS[2020]404).

## Conflict of Interest

The authors declare that the research was conducted in the absence of any commercial or financial relationships that could be construed as a potential conflict of interest.

## Publisher's Note

All claims expressed in this article are solely those of the authors and do not necessarily represent those of their affiliated organizations, or those of the publisher, the editors and the reviewers. Any product that may be evaluated in this article, or claim that may be made by its manufacturer, is not guaranteed or endorsed by the publisher.

## Abbreviations

UV: ultraviolet
LED: light-emitting diode
UV-B: ultraviolet-B
UVR8: UV RESISTANCE LOCUS 8
CRY: cryptochromes
PHY: phytochromes
COP: Constitutive Photomorphogenic
SPA: SUPPRESSOR OF PHYA
HY5: ELONGATED HYPOCOTYL5
PIF: phytochrome-interacting factor
WGCNA: weighted gene co-expression network analysis
RH: relative humidity
UPLC: ultra-performance liquid chromatography
GC-MS: gas chromatography–mass spectrometry
LC-MS: liquid chromatography–mass spectrometer
CTAB: cetyltrimethylammonium bromide
TPM: transcripts per million
DEGs: differentially expressed genes
FC: fold change
FDR: false discovery rate
GO: gene ontology
TOM: topological overlap matrix
qRT-PCR: quantitative real-time PCR
TAC: total anthocyanin content
SEMs: standard errors
BPC: base peak chromatogram
SCMs: significantly changed metabolites
Cy: cyanidin-3-*O*-glucoside
Dp: delphinidin-3-*O*-glucoside
Pn: peonidin-3-*O*-glucoside
Pt: petunidin-3-*O*-glucoside
Pl: pelargonidin 3-*O*-glucoside
Mv: malvidin-3-*O*-glucoside
PCA: principal component analysis
KEGG: Kyoto encyclopedia of genes and genomes
DEGs: differential expressed genes
GS: gene significance
MM: module membership
CWINV: cell wall invertase
FK: fructokinase
SD: standard deviation
C: control
B: blue light treatment
R: red light treatment
W: white light treatment
G: green light treatment
G-6-P: glucose-6-phosphate
F-6-P: fructose-6-phosphate.
