# Supplementary material for: Transcriptomic and Metabolomic Profiling Reveals the Effect of LED Light Quality on Fruit Ripening and Anthocyanin Accumulation in Cabernet Sauvignon Grape
Source: Front Nutr. 2021 Dec 14;8:790697. doi: 10.3389/fnut.2021.790697 (PMC8713590; doi:10.3389/fnut.2021.790697)
Supplement: Supplementary file 1 [file Data_Sheet_1.docx]

Fig. S1. Heatmap clustering showing correlation among 15 grape peel samples based on 891 metabolite (top 678 are pos and bottom 213 are neg) profiles. The color scale shows relative content from -3 to 3. Detailed information of samples, groups could be found in Table S2.


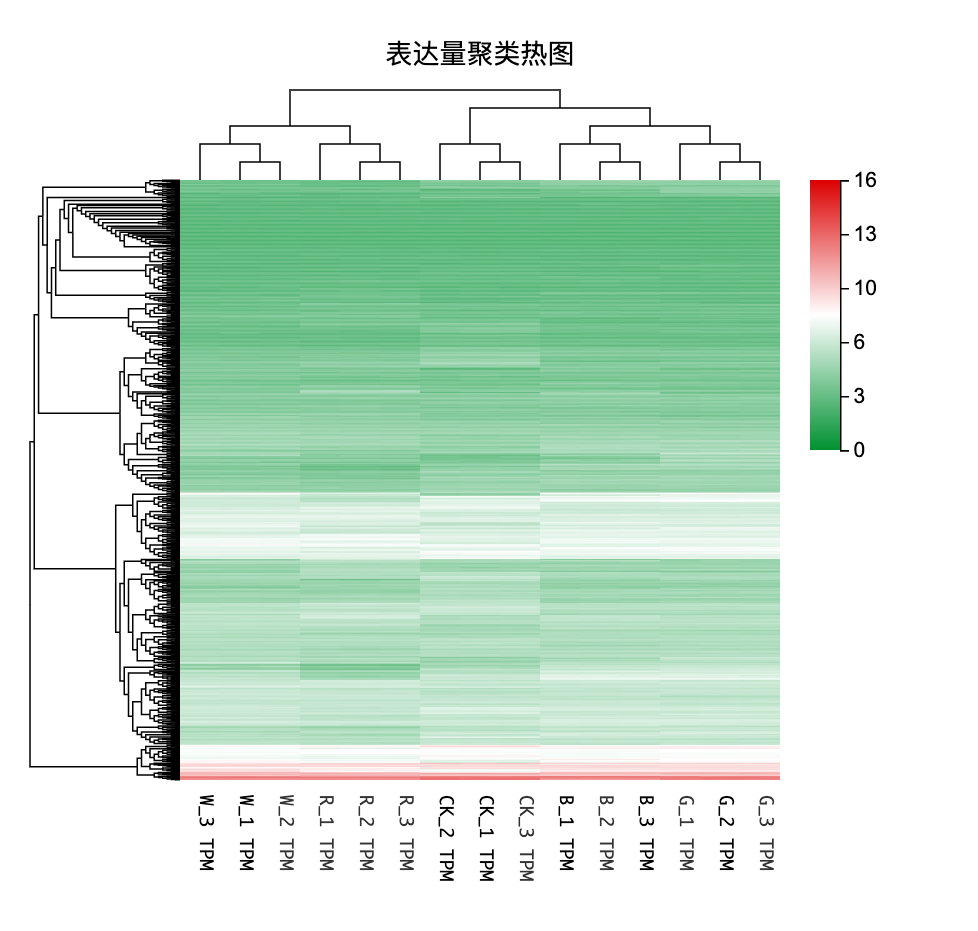


Fig. S2. Hierarchical cluster analysis of 23410 transcripts among 15 grape peel samples. The color scale shows relative content from 0 to 16.

Fig. S3. [GO](https://www.sciencedirect.com/topics/biochemistry-genetics-and-molecular-biology/gene-ontology) classification of DEGs. From left to right are C vs. B, C vs. W, C vs. R, C vs. G. C = Control (no light treatment), C: control (no light treatment), B: blue light treatment, R: red light treatment, W: white light treatment, G: green light treatment.

Fig. S4. Scale independence and mean connectivity by calculating 23410 genes. Correlation relation was set 0.85 and β was 10.

Fig. S5. Cluster dendrogram of 23410 genes (A), 27 module relationships of 23410 genes (B), the distribution of 8 modules with significantly related traits.


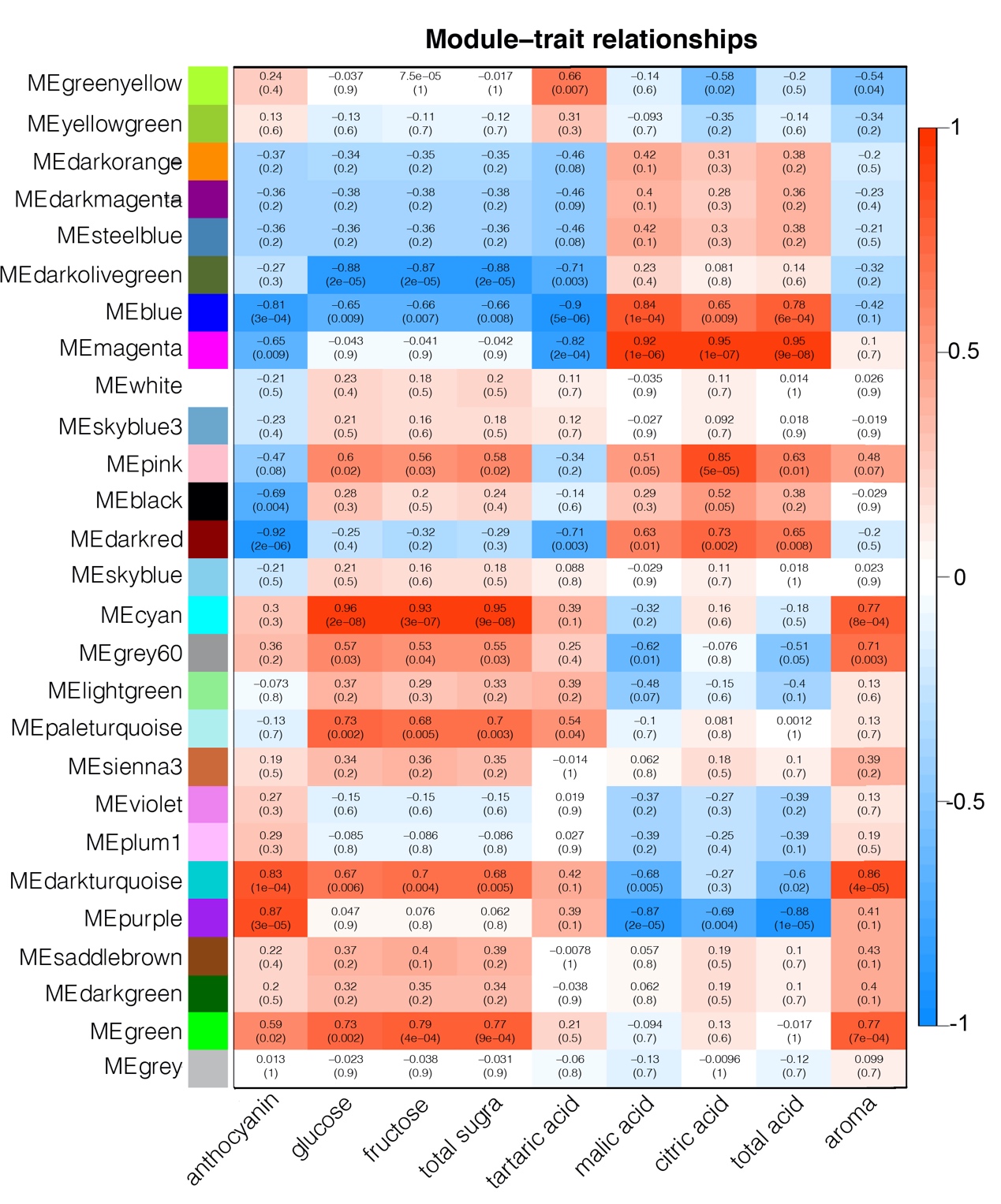


Fig. S6. The heat map of correlation between modules with anthocyanin, soluble sugar (glucose and fructose), organic acid (tartaric acid, malic acid, and citric), and aroma traits. The left panel shows the 27 modules. The color scale on right shows module trait correlation from 1 to −1.

Fig. S7. Employ qRT-PCR and metabolome to explain the differences in sugar and acid-related genes and metabolites in the flesh of grape berries after different light quality treatments. The heatmap shows the log_2_(FC) expression level of qRT-PCR between control and different light quality treatments, from left to right are white light, red light, blue light, and green light. The histogram represents the metabolites in the metabolome, from left to right represents under white light, green light, blue light, red light, and the control treatments. The panels in the upper right corner respectively indicate the amount of qRT-PCR (green to red). The histogram shows the content of metabolome substances. C: control (no light treatment), B: blue light treatment, R: red light treatment, W: white light treatment, G: green light treatment, G-6-P: glucose-6-phosphate, and F-6-P: fructose-6-phosphate.

Fig. S8. The qRT-PCR analysis of randomly selected transcripts in darkred modules. The columns indicate relative expression for the qRT-PCR (histogram) and RNA-seq (line chart). Correlation of gene expression between the qRT-PCR (x-axis) and the RNA-Seq (y-axis). The bars represent SD (n ≥ 3).

Fig. S9. The relative expression of light response and anthocyanin biosynthetic pathway genes in the skin after different light quality treatments. Values presented are the means ± SE (n=3). C: control (no light treatment), B: blue light treatment, R: red light treatment, W: white light treatment, G: green light treatment.

Fig. S10. The relative expression of genes in the flesh after different light quality treatments. Values presented are the means ± SE (n=3). C: control (no light treatment), B: blue light treatment, R: red light treatment, W: white light treatment, G: green light treatment.
