# Supplementary material for: Transcriptomic and Metabolomic Profiling Reveals the Effect of LED Light Quality on Fruit Ripening and Anthocyanin Accumulation in Cabernet Sauvignon Grape
Source: Front Nutr. 2021 Dec 14;8:790697. doi: 10.3389/fnut.2021.790697 (PMC8713590; doi:10.3389/fnut.2021.790697)
Supplement: Supplementary file 2 [file Data_Sheet_2.docx]

Table S1 Sequences of all primers used in the qRT-PCR

| Gene name | Gene ID | Forward sequence | Reverse sequence |
| --- | --- | --- | --- |
| Vv4CL | 100245991 | GAATGCTTCCAAGGCGAAAATC | GTGAAACCTTCTCCGATCTTTG |
| VvALMT1 | 100243329 | GTGTGTGGGAATTTGGAAAAGA | CTGTTGAATCCCCTGTTAAACG |
| VvALMT2 | 100250710 | TGTGAAGAAGTGGTGATCGTTA | GAAATCCATACTCATAAGGCGC |
| VvANS | 100233142 | GGACTACTTCTTCCACCTCATC | CTAGTCTCCCTTCTTCCAATCC |
| VvC4H | 100251539 | TTTCTACTCAATTTCCAAGCGC | AAAACCGGACCATACTTTTGTG |
| VvCHI | 100233078 | CAGGCAACTCCATTCTTTTC | TTCTCTATGACTGCATTCCC |
| VvCHS | 100263443 | AAACTATGTGCTACAGTCC | GACTACAGTTCAGAAATAA |
| VvCHX | 100264818 | CCTTCATGACAACTCCGATAGT | GAGTTGAGATTTCTTAGCGCTC |
| VvCOP1 | 100246063 | GGGACGTAAAGGAAAATATGCC | CAGGCAATGTATTCGCTGTTTA |
| VvCOP2 | 100248310 | TCAATGACCTCCAACAGTGTTA | TGGTATTCCTTTCATCCTGCTT |
| VvCS1 | 100255795 | GTGATGCGGGGATTCTTAGATA | TCCCATACATTAAGAGGTACGC |
| VvCS2 | 100261432 | TCTCAGTTAATATGGGACCGTG | GAGTTTTAGAATTAGGCGGCTG |
| VvDFR | 100233141 | CTTTCATTTTCTTCCCCGGATC | CATCGAAACTTCCTTCATCAGC |
| VvECAR | 100257265 | ATGGCTATACTC ATTCTGGGAC | ATCTCTCCAAGCAAGCAGCA |
| VvEGS | 100853950 | AGTATTACCGTCTATGGCAG | TTCACAACCTCTTCCTCTG |
| VvF35'H1 | 100232896 | GATCAACATGGACGAAGCTTTC | AGCCACAGGAAACTAAAACAAC |
| VvF35'H2 | 100261319 | ATTTTGGGATTTTTGCCTGTGG | CATAGATGCATATGGCTTGGTG |
| VvF3H | 100233079 | CCAATCATAGCAGACTGT CC | TCAGAGGATACACGGTTGCC |
| VvF3'H | 100232999 | GATGTTAGGCAGGAGAGTGTTC | ATGGAGCTTCTTCATCTTGGAA |
| VvFK1 | 100250938 | GTGAGTTCATGTTCTACCGGAA | TCAAGCTTATGGATCCGTAGTG |
| VvFX2 | 100258640 | GCATATCGAAATTGGGAGGTTC | TTCATGGAAAAGCATATCAGCG |
| VvGPI1 | 100252335 | CTTAATTCAGCTCGAACGAAGG | GTTGCGGATTATGGATTAGCAG |
| VvGPI2 | 100262681 | GACATTCATTGAAGTTCTGCGT | ATATTCCAACTGCTCGCTCATA |
| VvGST1 | 100243577 | TCGAGCTATGTATCAAGCCAAT | CTCGTACACATCCAAAACCTTG |
| VvGST2 | 100265903 | CCAAGCTCTGTTACGAGCTATT | CTCGTACACATCCAAAACCTTG |
| VvHXX1 | 100242358 | GGAACAAATGCAGCCTATGTAG | GCGTACAATGTCTCCCAAATAC |
| VvHXX2 | 100255753 | CCGTTCTTCACTCTTTTCTTCC | AATGAGTCAAAATTGGAGCGAC |
| VvHY5 | 100261472 | CACCACCACAACAACAAATACT | GGACAAGTTTGATCATCAGCTC |
| VvHYH | 104879018 | GTCAAAGACTTGGAGAGGAAGA | TCTCTTTCTCTCACAAAGACCC |
| VvIV1 | 100232951 | CAAAAACCTTCTTTGACAACGC | CAGACCATCCCTTCTCGATATC |
| VvIV2 | 100256970 | GAAGATGTTTTACTCGAACCCG | TCGCTTCTAAGGTCTCGTTATC |
| VvMDH1 | 100232859 | AGGGAGCAGATTTGGTGATTAT | GCATTGATGTTGAACAGATCGT |
| VvMDH2 | 100248145 | GCAATTATCAAAGCACGAAAGC | GAAGGAATAGATAAGCCCTGCT |
| VvME1 | 100233075 | ATACATTTTCCCTGGATTCGGT | TGGGTATATCATTCCCTTGTCG |
| VvME2 | 100233140 | AGTTATTGTCGTCACTGATGGT | TCTCCGTTGTTTGAGACCAATA |
| VvMYB5A | 100233122 | CGATAGCAATGAGAATTGGCAA | GAAGAGAATGTATCGTCGTTGC |
| VvMYB5B | 100232973 | CAGTGCCAATATCCAGAATTCG | GTTGTCCAGGAAACAAATCCTC |
| VvMYB90 | 100233098 | GCTCAATTATTTGAAGCCGGAT | CTGAACCTCCTTTTTGAAGTGG |
| VvPAL | 100241575 | CTGATCAGATTCTTGAACGCTG | ATCTGATGCCTGAGTATCCTTG |
| VvPEPC | 100256754 | AGGGCTTATCATTCACTGTTCA | GTAGAGGATGTCATGTAGTGGG |
| VvPIF3.1 | 100247781 | CTCTCTTTTGGACCTGAAAACG | TCCAGTATCTTTATCACGAGGC |
| VvPIF3.2 | 100253874 | TTTGTCTGGAATAGCAGGAACT | GAAGGGTGCACTTGATAATGAC |
| VvPIF4 | 100262490 | ACAATTGGTTCATTTCACGAGG | TCTTCTGATTGGTTACAGGGAC |
| VvPK1 | 100255934 | ATCTTGTCCATGTTCGTAAGGT | GCCTACCAGGTTACACTTGTAT |
| VvPK2 | 100261276 | TGCAACAAATGAGTCAGTTCTG | ACAACTCGGTCATGTGACTTTA |
| VvQR | 100852555 | GTGATAAGGCAGTGAAGGTA | CCTCTCCGTTTGAAGTTACT |
| VvSPA1 | 100256812 | ACCGTGATAAGTTGACTCAAGT | CTTCAAACTTACCATAGCGAGC |
| VvSPA2 | 100257085 | AGAGATAGAATGCAGATCAGGC | GGGAAAGATGAGTATCCCACAT |
| VvSPP | 100258115 | ACTATTGGAAAAACGTGGGTTG | CCAGCACCTTGGGGTAATATAT |
| VvSPS1 | 100232974 | GACAGTGACCTCAAGTCCTTAA | CATTTTTCTTAGGATCTGGCCG |
| VvSPS2 | 100241955 | AAAGAACTTCTATGGCCGTACA | GGACATTTAAAGCGCCAGATAG |
| VvSS1 | 100267606 | GCTTCAAAGAAGAGCTTGTTGA | GAGATGACGGTTGAGAAATTGG |
| VvSS2 | 100249279 | CAAGCAGCAAGGACTTGATATC | GGATGATTGAATGCTCTGTTCC |
| VvUFGT | 100233099 | ATTTGGTTTGCTGCAGATATGG | CGCGAAAACGTACTTTAGACAT |
| VvUBQ | 100241514 | GAGGGTCGTCAGGATTTGGA | GCCCTGCACTTACCATCTTTAAG |

Table S2 Differentially anthocyanins in grape skin after different light quality treatment.

| Component name | Anthocyanins content/(mg.kg^−1^) FW of different treatment | | | | |
| --- | --- | --- | --- | --- | --- |
|  | W | G | C | B | R |
| Cy 3-O-glu | 0.135 ± 0.032 b | nd | nd | 0.883 ± 0.126 a | nd |
| Cy 3-O-(6''-acetyl-glu) | nd | nd | nd | 0.984 ± 0.102 | nd |
| Cy 3-O-(6''-caffeoyl-glu) | nd | nd | nd | nd | nd |
| Cy 3-O-(6''-p-coumaroyl-glu) | 0.324 ± 0.079 b | nd | nd | 0.717 ± 0.126 a | nd |
| Cy 3-O-caffeoylglucoside-5-O-glu | 0.755 ± 0.135 | nd | nd | nd | nd |
| Cy 3-O-coumaroylglucoside-5-O-glu | 0.193 ± 0.042 | nd | nd | nd | nd |
| Cy 3-O-feruloyl-glu | nd | nd | nd | nd | nd |
| Cy 3-O-glucosyl-glu | 0.899 ± 0.214 | nd | nd | nd | nd |
| Dp 3-O-glu | 0.612 ± 0.192 b | nd | nd | 2.263 ± 0.428 a | nd |
| Dp 3,5-O-diglucoside | 0.855 ± 0.233 a | nd | nd | 0.277 ± 0.042 b | nd |
| Dp 3-O-(6''-acetyl-glu) | 0.173 ± 0.024 | nd | nd | nd | nd |
| Dp 3-O-(6''-caffeoyl-glu) | 0.189 ± 0.011 a | nd | nd | 0.178 ± 0.021 a | nd |
| Dp 3-O-(6''-p-coumaroyl-glu) | 0.340 ± 0.045 a | 0.127 ± 0.035 b | nd | 0.122 ± 0.046 b | nd |
| Dp 3-O-acetylglucoside-5-O-glu | nd | nd | nd | 0.179 ± 0.024 | nd |
| Dp 3-O-glucosyl-glu | nd | nd | nd | 0.138 ± 0.019 | nd |
| Mv 3-O-glu | 5.968 ± 0.557 b | 6.644 ± 0.623 b | 0.588 ± 0.108 c | 18.234 ± 2.148 a | 0.125 ± 0.029 d |
| Mv 3,5-O-diglucoside | nd | nd | nd | 0.180 ± 0.072 | nd |
| Mv 3-O-(6''-acetyl-glu) | 0.362 ± 0.052 | nd | 0.266 ± 0.046 | nd | 0.242 ± 0.028 |
| Mv 3-O-(6''-p-coumaroyl-glu) | 2.583 ± 0.412 b | 5.323 ± 0.917 a | 0.425 ± 0.092 c | 4.922 ± 1.198 a | 0.250 ± 0.023 d |
| Mv 3-O-acetylglucoside-5-O-glu | 0.535 ± 0.132 | nd | nd | nd | nd |
| Mv 3-O-caffeoylglucoside-5-O-glu | 0.856 ± 0.198 | nd | nd | nd | nd |
| Mv 3-O-coumaroylglucoside-5-O-glu | 0.570 ± 0.105 | nd | nd | nd | nd |
| Pl 3-O-glu | nd | nd | nd | 0.143 ± 0.049 | nd |
| Pn 3-O-glu | nd | 0.676 ± 0.177 b | 0.163 ± 0.025 c | 3.565 ± 0.105 a | 0.489 ± 0.098 b |
| Pn 3,5-O-diglucoside | 0.212 ± 0.045 | nd | nd | nd | nd |
| Pn 3-O-(6''-acetyl-glu) | 0.184 ± 0.038 c | 0.559 ± 0.124 b | 0.189 ± 0.035 c | 1.487 ± 0.218 a | 0.461 ± 0.144 b |
| Pn 3-O-(6''-caffeoyl-glu) | nd | 0.886 ± 0.218 | nd | nd | nd |
| Pn 3-O-coumaroylglucoside-5-O-glu | 0.182 ± 0.029 | nd | nd | nd | nd |
| Pt 3-O-glu | nd | 0.143 ± 0.018 | nd | nd | nd |
| Pt 3,5-O-diglucoside | 0.216 ± 0.041 | nd | nd | nd | nd |
| Pt 3-O-(6''-acetyl-glu) | 0.376 ± 0.039 a | 0.126 ± 0.029 b | nd | nd | nd |
| Pt 3-O-(6''-caffeoyl-glu) | 0.743 ± 0.192 a | nd | nd | 0.180 ± 0.033 b | nd |
| Pt 3-O-(6''-p-coumaroyl-glu) | 0.862 ± 0.133 a | 0.838 ± 0.145 a | nd | 0.257 ± 0.062 b | 0.192 ± 0.055 b |
| Pt 3-O-caffeoylglucoside-5-O-glu | 0.169 ± 0.038 | nd | nd | nd | nd |
| Pt 3-O-coumaroylglucoside-5-O-glu | 0.895 ± 0.127 | nd | nd | nd | nd |

Note: Cy, Cyanidin, Dp, Delphinidin, Mv, Malvidin, Pl, Pelargonidin 3-O-glucoside, Pn, Peonidin, Pt, Petunidin, nd, not detected, glu, glucoside. A significance level of p < 0.01 was applied.
